# Study on Soot and NOx Formation Characteristics in Ammonia/Ethylene Laminar Co-Flow Diffusion Flame

**DOI:** 10.3390/molecules29174003

**Published:** 2024-08-24

**Authors:** Shuanglong Li, Qianqian Liu, Feng Zhang, Jingyun Sun, Yang Wang, Mingyan Gu

**Affiliations:** School of Energy and Environment, Anhui University of Technology, Ma’anshan 243002, China; a919212091@163.com (S.L.); liuqianqian208dw@163.com (Q.L.); 15955014750@163.com (F.Z.); jingyunsun133@163.com (J.S.); wangyang@ahut.edu.cn (Y.W.)

**Keywords:** ammonia–ethylene co-combustion, co-flow diffusion flame, soot, NOx

## Abstract

The formation of soot and NOx in ammonia/ethylene flames with varying ammonia ratios was investigated through experimental and numerical analysis. The spatial distribution of the soot volume fraction and NOx concentrations along the flame central line were measured, and the mechanism of soot and NOx formation during ammonia/ethylene co-combustion was analyzed using CHEMKIN 17.0. The experimental results indicated that the soot volume fraction decreases with an increase in ammonia ratio, with the soot peak concentration occurring in the upper region of the flame. The distribution of NOx is complex. In the initial part of the flame, a higher concentration of NOx is generated, and the lower the ammonia ratio, the higher the concentration of NOx. As the combustion process progresses, the concentration of NOx initially decreases and then subsequently increases rapidly, with higher ammonia ratios leading to higher concentrations of NOx. The addition of ammonia results in a decrease in CH_3_, C_2_H_2_, and C_3_H_3_, and an increase in CN concentration. This leads to a transformation of carbon atoms within the combustion system, reducing the available carbon for soot formation and suppressing its generation. A higher ammonia ratio increases the likelihood that NH_3_ will be oxidized to N_2_, as well as increasing the probability that any generated NO will undergo reduction to N_2_ through the action of the free radicals NH_2_ and NH.

## 1. Introduction

With the increasing severity of energy shortages and global warming issues, ammonia is considered as a potential renewable energy source and fuel for the future [1,2,3]. Only a small amount of carbon dioxide is produced during ammonia synthesis [4,5], and no carbon dioxide is emitted during combustion [6]. This is of great significance for the development of social production and the promotion of global carbon neutrality [7]. However, in comparison to hydrocarbon fuels, ammonia does have certain drawbacks as a source of energy [8]. The calorific value of ammonia combustion is about 40% that of methane and propane combustion, while the maximum laminar combustion velocity of ammonia/air flame is about 20% that of methane and propane [9]. In addition, the ignition limits and temperature of a pure ammonia/air mixture are narrow and high, respectively, indicating that ammonia has low combustibility [10]. Moreover, due to the lack of CO_2_ in the combustion products [11], the flame temperature of pure ammonia/air flame is lower [12] and the heat radiation transfer from the flame is less than that of hydrocarbon flames [9,13]. Another challenge of pure ammonia combustion is the high emission of nitrogen oxides (NOx) after combustion. To increase the combustion intensity of ammonia, the mechanism of ammonia oxidation has attracted much attention [14] and co-combustion of ammonia with various hydrocarbon fuels has attracted people’s attention [15,16,17,18]. However, the unavoidable generation of soot and NOx during this process remains a concern [19,20,21]. Therefore, it is crucial to investigate the characteristics of soot and NO produced during the combustion process of hydrocarbon fuels mixed with ammonia. This research is necessary in order to effectively control the emission of pollutants from the combustion of hydrocarbon fuels mixed with ammonia.

Bennett et al. [22] studied the effect of ammonia addition on the soot volume fraction in ethylene opposed diffusion flames. The results showed that the soot volume fraction in ethylene flames was significantly reduced after ammonia was added. In addition, Zhou et al. [23] measured the change in the soot volume fraction in ethylene flames after nitrogen and ammonia were added, indicating that the chemical effect of ammonia played a major role in inhibiting the soot formation of ethylene flames. Furthermore, Steinmetz et al. [24] compared the effect of hydrogen and ammonia addition on methane and ethylene diffusion flame soot formation. The results indicated that the chemical effect of hydrogen promoted soot formation, while the chemical effect of ammonia inhibited soot formation. Moreover, Liu et al. [25] studied the characteristics of soot formation in ethylene flames with ammonia addition and found that the inhibitory effect of ammonia on substances with more aromatic rings is greater. Ren et al. [26] simulated the diffusion flames of ammonia/ethylene using CHEMKIN 17.0, which showed that ammonia participates in the chemical reactions during combustion, leading to a decrease in the concentrations of C_2_H_2_, C_3_H_3_, P-C_3_H_4,_ and C_4_H_4_, thereby inhibiting the formation and growth of PAHs. In addition, Boyette et al. [27] studied the effect of compositional inhomogeneity between ammonia and ethylene on soot formation characteristics. The simulation results indicated that flames with higher premixed degrees exhibit greater A4 concentrations. Zhang et al. [28] used the CoFlame code to study the effect of ammonia addition on the soot inception, growth, and oxidation processes of ethylene flames. The results showed that the addition of ammonia reduced the soot inception, growth and oxidation rate. Adding ammonia increased the consumption path of H radicals and reduced the reaction rate of H radical generation, thereby inhibiting the rate of the HACA reaction.

Despite NH_3_ addition having significant reduction effects on soot, increased NO emission is a major penalty for NH_3_ addition in hydrocarbons. Various models have been proposed to study the effect of NH_3_ addition on NO formation. Rocha et al. [29] investigated chemical kinetic modelling of ammonia/hydrogen/air ignition, premixed flame propagation, and NO emissions. The results showed that pure NH_3_ flames had high ignition delay times and low flame speeds, and that the addition of H_2_ to the NH_3_ flame increased the flame speed exponentially and significantly increased NOx emissions. Pathway analyses were conducted for NO formation in counterflow premixed NH_3_/CH_4_/air flames [30] and showed that the main precursors of NO were HNO, NH_2_, and CH_3_. Abián et al. [31] investigated the effect of the main nitrogen oxides (NO, NO_2_, and N_2_O) present in combustion systems on soot and the main product gases formed from the pyrolysis of ethylene. The experimental findings revealed that the presence of nitrogen oxides affects soot formation, with the lowest tendency for sooting observed in the presence of NO_2_, followed by NO and then N_2_O. Additionally, different mechanisms appear to play a role in reducing both soot and nitrogen oxides, including oxidation and reburn type reactions [31,32,33]. Montgomery et al. [34] investigated the effect of ammonia on soot formation in methane co-current diffusion flames. They measured soot volume fractions and mole fractions of gas-phase species, and further performed chemical kinetic simulation, which showed that the chemical inhibition of soot formation in hydrocarbon flames by ammonia is related to the NOx produced during its oxidation. Guo et al. [35] conducted a numerical investigation on the interaction between soot and NO formation in a laminar axisymmetric co-flow ethylene/air diffusion flame. The results indicate that the formation of NO has minimal impact on soot formation, while the presence of soot in the flame significantly suppresses NO formation. Specifically, the peak NO concentration and NO emission index are reduced by 28% and 46%, respectively, due to the presence of soot. This influence is attributed to both radiation-induced thermal effects and reaction-induced chemical effects. The thermal effect accounts for a reduction of 25% in peak NO concentration and 38% in NO emission index, while the chemical effect arises from competition for acetylene (C_2_H_2_) between soot and NO formation. Soot consumption of acetylene leads to lower radical CH formation rates, subsequently reducing the reaction rate of CH + N_2_ = HCN + N, which is the rate-limiting step in the prompt NO formation route.

The aforementioned study suggests that soot and NOx are the two primary pollutants formed during the combustion of hydrocarbon fuels. However, there is still a lack of corresponding research on the characteristics and interaction mechanisms of soot and NO produced in ammonia co-firing flames. This paper presents a study using a laminar co-flow diffusion flame platform to measure the spatial distribution of the soot volume fraction and the variation in NOx concentrations in ammonia/ethylene flames with different ammonia addition conditions. The formation processes of soot and NOx are analyzed using the CHEMKIN 17.0 software with a combined chemical mechanism.

## 2. Results and Discussion

### 2.1. Experimental Results and Discussion

#### 2.1.1. SVF Results and Analysis

**Figure 1:**
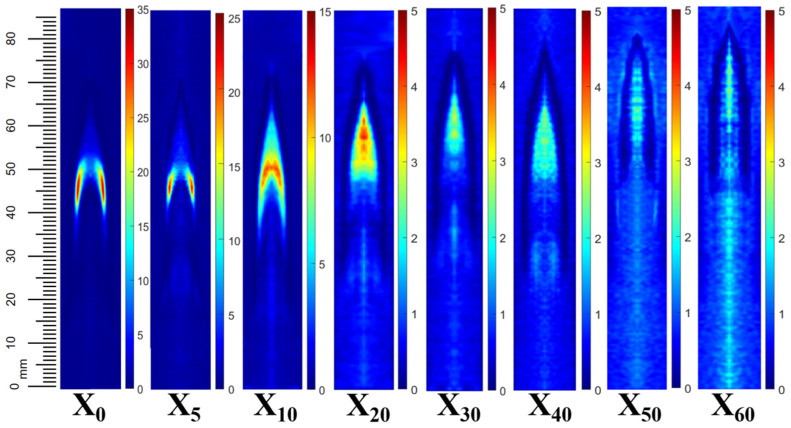
Two-dimensional distribution of soot volume fraction in X_0_–X_60_ flame.

Figure 1 shows the soot volume fraction in X_0_–X_60_ flames measured by the light extinction method. The maximum soot volume fraction in each flame is 33.21, 22.26, 12.03, 4.34, 3.41, 3.07, 2.68, and 2.43 ppm, respectively. After adding ammonia, a decrease in the soot volume fraction in the flame can be observed. In the pure ethylene co-flow diffusion flame, the maximum soot concentration region is typically located near the two sides of the flame (flame wings). However, as the content of ammonia increases, it moves towards the flame centerline, indicating that the addition of ammonia does affect the overall structure of the C_2_H_4_ base flame, in agreement with the study of Ren et al. [26]. Ref. [36] revealing that that visible C_2_H_4_ co-flow diffusion flame height is largely lifted off as ammonia content increases.

**Figure 2:**
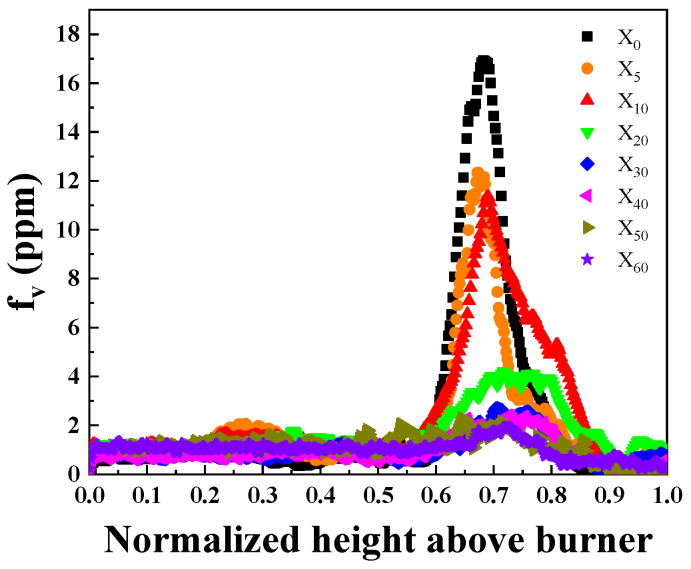
The distribution of the soot volume fraction along the centerline of the flame with different ammonia addition ratios.

Figure 2 shows the distribution of the soot volume fraction along the centerline of the flame under different ammonia addition ratios. In order to more accurately compare the effect of ammonia addition on the soot formation of ammonia/ethylene flames, the normalized height above burner (NHAB) was adopted to eliminate the impact of variations in flame height on the analysis of the results. It can be seen from the figure that the soot volume fraction at the centerline of the flame decreases with ammonia addition. For the cases with larger ammonia addition ratios, the position of the maximum soot volume fraction at the centerline shifts upwards.

In order to obtain the total soot volume fraction change of the flames, *F_v_* is used to express the total amount of soot volume fraction in the flame on the cross-section of each height of the flame, and the integral value of the soot volume fraction at the z flame height was calculated using the following formula [37]:

(1) $$F_{v}\left(z\right)=2\pi \int_{0}^{r_{\left(z\right)}}f_{v}\left(i,z\right)idi$$

where *f_V_ (i*, *z*) is the soot volume fraction at r (*i*), and r (*z*) is the radius of the flame at the z flame height. When the amount of the soot volume fraction at each height interface of the flame is F*_v_*, the total yield of soot in the flame Φ is obtained by integrating the *F_v_* of each height of the flame along the axial flame height; the calculation formula is [38,39]:

(2) $$\Phi =\int_{0}^{h_{f}}F_{v}dz$$

where *h_f_* is the visible height of the flame and *z* is the height of a point in the flame.

**Figure 3:**
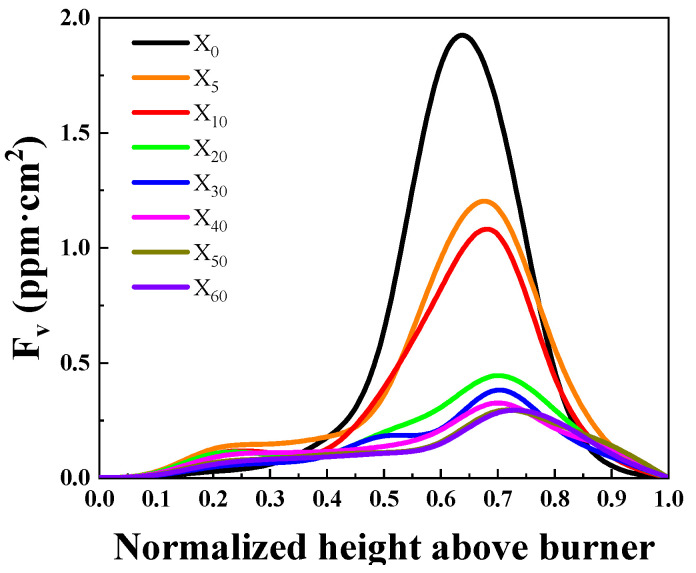
Soot distribution at different ammonia mixing ratios.

Figure 3 shows the overall change of soot concentration in the flame for X_0_–X_60_ conditions. From the figure, it can be seen that the total amount of soot in the cross-section is significantly reduced after ammonia mixing, and the maximum value of the total amount of the cross-section is shifted to the downstream of the flame. Initially, the maximum value of the total soot in the X_0_ flame appeared at the height of 0.6–0.7, and then, with the increase in the proportion of ammonia, the maximum value appeared moving to the right, and the maximum value of the X_60_ flame appeared at the height of 0.7–0.8.

#### 2.1.2. NOx Results and Analysis

**Figure 4:**
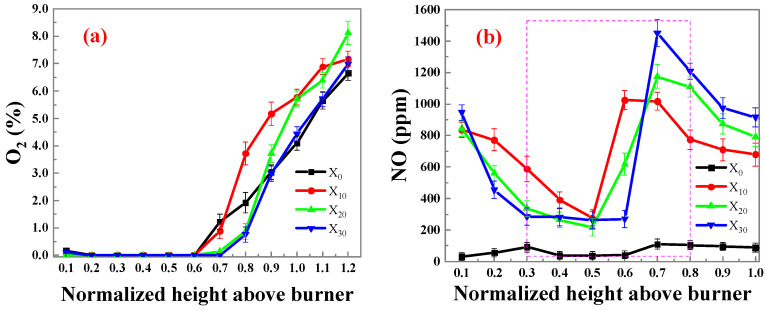
Variation of (**a**) O_2_ and (**b**) NOx with different ammonia mixing ratios.

Figure 4 shows the distribution of O_2_ and NOx concentration under different ammonia mixing ratios. From Figure 4a, it can be seen that before NHAB = 0.6, the oxygen content at these heights is close to 0. This is due to the fact that around this zone, there is not enough oxygen diffused from the outside to the flame center zone, leaving much fuel there, including the cracked species, which results in a low-oxygen zone. When NHAB > 0.6, as the fuel is consumed, external oxygen diffuses towards the center, leading to an increase in the concentration of oxygen. The addition of ammonia increases the combustible gas in the fuel, resulting in a longer combustion process and a longer low-oxygen concentration zone.

Figure 4b shows the variation in NOx concentration in the flame. From Figure 4b, it can be seen that, at the base zone of the flame, a significant amount of hydrocarbon radicals and amine substances are produced as a result of ethylene and ammonia cracking, leading to a reduction in generated NOx, and the higher the ratio of ammonia, the lower the concentration of NOx. In the middle section of the flame, a zone with high temperature and low oxygen concentration is formed due to the intense reaction, which is not conducive to NOx generation. Additionally, both ammonia and ethylene have a reducing effect on NOx. Simultaneously, soot may also play a role in NOx reduction. At the upper part of the flame, external oxygen diffuses into the flame, leading to an increase in the generation of high levels of NOx. Additionally, a higher ammonia ratio results in more NOx.

In summary, the addition of NH_3_ has a significant impact on reducing soot and NOx emissions. It is important to note that when the ammonia doping exceeds 20%, the inhibitory effect on soot gradually weakens, and NOx emissions increase linearly with the ammonia doping ratio.

### 2.2. Numerical Results and Discussion

#### 2.2.1. The Effect of Ammonia on Soot Formation

##### PAHs Generation Concentration Analysis

To evaluate the variations in the formation of soot precursors pre- and post-addition of ammonia, a set of four aromatic hydrocarbons, namely benzene (A1), naphthalene (A2), phenanthrene (A3), pyrene (A4), and coronene (A7) were chosen for analysis.

**Figure 5:**
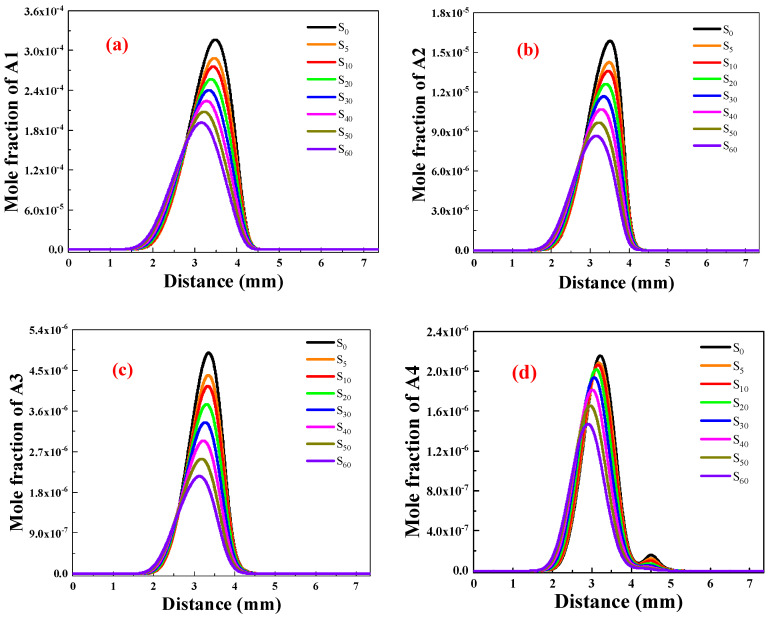
Distribution of A1-A4 concentrations after adding different proportions of ammonia. (**a**) A1; (**b**) A2; (**c**) A3; (**d**) A4.

Figure 5a shows the distribution of A1 concentrations for different ammonia ratios and the changes after normalizing maximum A1 concentrations. The figure indicates a gradual decrease in A1 concentration as the proportion of ammonia increases. Compared to the flame without ammonia, ammonia addition results in an 8.97%, 13.00%, 19.01%, 24.27%, 29.40%, 34.54%, and 37.75% decrease in A1 concentrations. At distances of 1–2.5 mm, A1 concentration is higher than without ammonia due to reduced formation caused by ammonia, leading to delayed generation at the fuel side and higher overall concentration compared to that without ammonia.

Figure 5b illustrates the A2 concentration distribution after adding different amounts of ammonia. Similarly to A1, the maximum A2 concentration decreases as more ammonia is added. Compared to the S0 flame without ammonia, the A2 concentration for S5–S60 decreased by 10.02%, 14.30%, 20.54%, 26.46%, 32.64%, 38.96%, and 45.23%, respectively, indicating that ammonia has a stronger inhibitory effect on A2 than on A1 due to the necessary participation of A1 in generating A2 and its inability to be fully converted into A2.

Figure 5c shows that A3 concentration decreases as ammonia proportion increases. Compared to the S0 flame without ammonia, A3 concentration in S5–S60 decreased by 10.18%, 15.24%, 23.36%, 31.58%, 39.93%, 48.11%, and 55.99%, respectively, indicating that ammonia has a stronger inhibitory effect on A3 formation than A1 and A2 due to its suppression of A2 production, which is the primary precursor of A3. This leads to a more significant inhibitory effect on A3 production by ammonia.

Figure 5d shows the distribution of A4 concentrations, indicating that the addition of ammonia inhibits A4 production. The inhibitory effect becomes more significant with an increased proportion of ammonia, as evidenced by the reduction in A3 concentration in flames containing ammonia. Despite the augmented pathway of A4 generation, sufficient ammonia leads to a significant inhibition of A4 production by affecting the reaction substrates in the A4 production pathway.

As shown in Figure 5a–d, the NH_3_ addition significantly lowers the A1–A4 mole fractions in C_2_H_4_ flame, indicating the chemical effect of ammonia on suppressing soot precursor formation. The experimental studies of Bennett et al. [22] have found that the mole fraction of C_2_H_2_, C_3_H_3_, PC_3_H_4_, and C_4_H_4_, which are responsible for the first aromatic benzene ring formation and larger PAH growth, were substantially affected by the addition of ammonia. Ammonia addition reduced the formation of these key radicals, leading to a decreasing concentration of A1–A4 and a reduction in the soot volume fraction.

##### ROP Analysis of A1

To investigate the impact of ammonia addition on polycyclic aromatic hydrocarbons production, this section focuses on analyzing the rate of product (ROP) for the first aromatic ring A1. A1 is the initial aromatic ring created in a non-aromatic fuel flame and its production reaction serves as the foundation for ensuing PAH growth and soot formation. Therefore, understanding the reaction mechanism of A1 production is crucial for studying soot formation.

**Figure 6:**
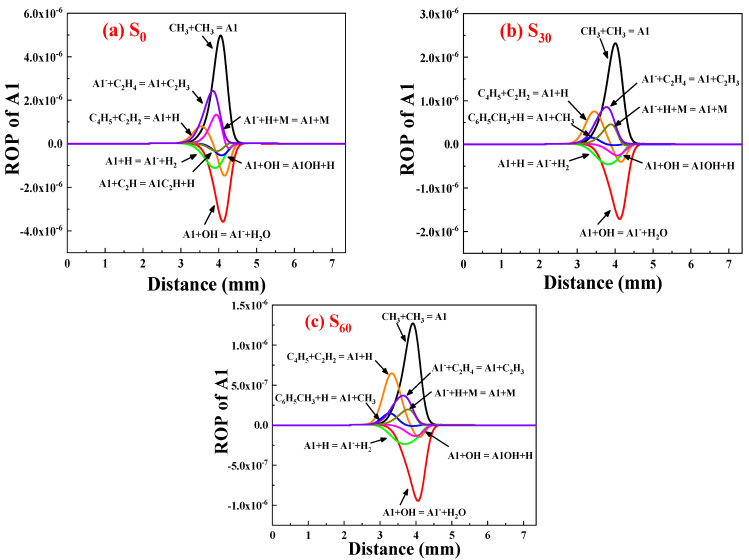
ROP of A1 after adding ammonia: (**a**) 0%; (**b**) 30%; (**c**) 60%.

Figure 6 shows the distribution of reaction rates for A1 with 0%, 30%, and 60% ammonia. The formula and magnitude of the first eight digits of the reaction rate are presented, with positive values indicating production of A1 and negative values indicating consumption. The fastest reaction producing A1 is the methyl addition: C_3_H_3_ + C_3_H_3_ = A1, regardless of ammonia presence. The quickest consumption of A1 occurs in the oxidation reaction: A1 + OH = A1^−^ + H_2_O.

The addition of 30% and 60% ammonia significantly reduces the reaction rate of A1 production by more than 50% and 75%, respectively, leading to a significant inhibition of A1 production. This reduction leads to a decreased formation of crucial products for the subsequent growth of PAHs through the HACA process. Specifically, the decreased rate of reaction consuming A1 results in reduced formation of A1^−^.

As a result, the subsequent formation of larger polycyclic aromatic hydrocarbons is impeded. It is noteworthy that, in the first eight positions of the reaction rate of A1 after the addition of ammonia, the reaction rate of the A1 consumption reaction A1 + C_2_H = A1C_2_H + H decreased, while the rate of the A1 generation reaction C_6_H_5_CH_3_ + H = A1 + CH_3_ increased. However, the change in reaction rate has negligible effects compared to the reaction C_3_H_3_ + C_3_H_3_ = A1, which dominates A1 formation and exerts a greater influence on a decrease in the concentration of A1 after ammonia addition.

##### Sensitivity Analysis of A1

**Figure 7:**
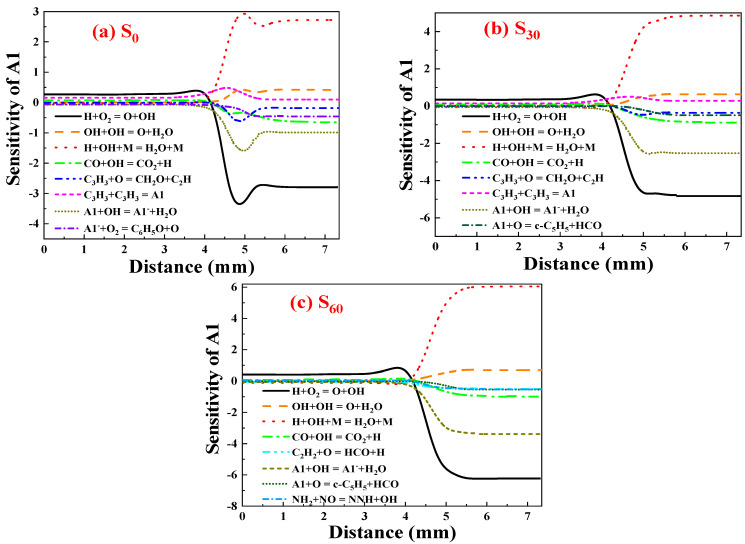
Sensitivity coefficient of A1 after adding (**a**) 0%, (**b**) 30%, and (**c**) 60% ammonia.

Figure 7 shows the sensitivity coefficient of A1 to ammonia addition. Positive values indicate A1 formation, while negative values indicate A1 consumption. The reaction H + OH + M = H_2_O + M is critical for A1 formation at 4.5–7.35 mm due to strong oxidizing substances like O_2_ and OH on the oxidant side. As distance from the fuel port decreases below 4.5 mm, the sensitivity coefficient approaches 0, indicating reduced effect of H and OH on A1 formation due to decreased concentration of oxidizing substances towards the fuel side.

The reaction OH + OH = O + H_2_O is also beneficial to the formation of A1 as it consumes more oxidizing substances, thus slowing down A1 consumption. The reaction C_3_H_3_ + C_3_H_3_ = A1 is also advantageous for A1 production by directly producing A1. Among all the reactions favoring A1 consumption, H + O_2_ = O + OH has the largest absolute sensitivity coefficient at 4.5–7.35 mm, indicating the most critical consumption of A1. This is because A1 is still undergoing the generation stage with high concentrations of O and OH radicals, which enable the oxidation of reactants involved in the formation of A1. The reactions A1 + OH = A1^−^ + H_2_O and A1^−^ + O_2_ = C_6_H_5_O + O also demonstrate increasing absolute sensitivity coefficients (both with negative values). In this process, OH oxidizes A1 to A1^−^, which is further oxidized by O_2_ to form C6H_5_O. Thus, at 4.5–7.35 mm, the oxidation of A1 dominates its consumption.

At 0–4.5 mm, the most favorable reaction for the formation of A1 is H + O_2_ = O + OH. As in the fuel-rich region, oxidants like O and OH function to activate the reaction substances involved in A1 production, favoring the production of A1. When 30% ammonia is added, H decreases, making the sensitivity coefficient of H + OH + M = H_2_O + M greater at 4.5–7.35 mm compared to when ammonia is absent, influencing A1 formation further. Also, due to the decrease in H, the absolute value of the sensitivity coefficient of the H + O_2_ = O + OH reaction increases. When H decreases, this reaction produces more O and OH, thus enhancing its effect on A1 consumption. Due to an increase in substances to be activated on the fuel side following the addition of ammonia and the decrease in H, the sensitivity coefficient of H + O_2_ = O + OH increases below 4.5 mm. This indicates that the reaction is more critical for A1 formation.

Before the addition of ammonia, the oxidation reaction of A1 involves A1 reacting with OH to form A1^−^, which is further oxidized to form C_6_H_5_O. Following the addition of 30% ammonia, the direct oxidation reaction of A1, A1 + O = c-C_5_H_5_ + HCO, exhibits increased sensitivity, thus becoming more important for the consumption of A1. When 60% of ammonia is added, OH decreases in the flame; the reaction NH_2_ + NO = NNH + OH will play a more critical role in the consumption of A1.

##### Reaction Path Analysis of A1

**Figure 8:**
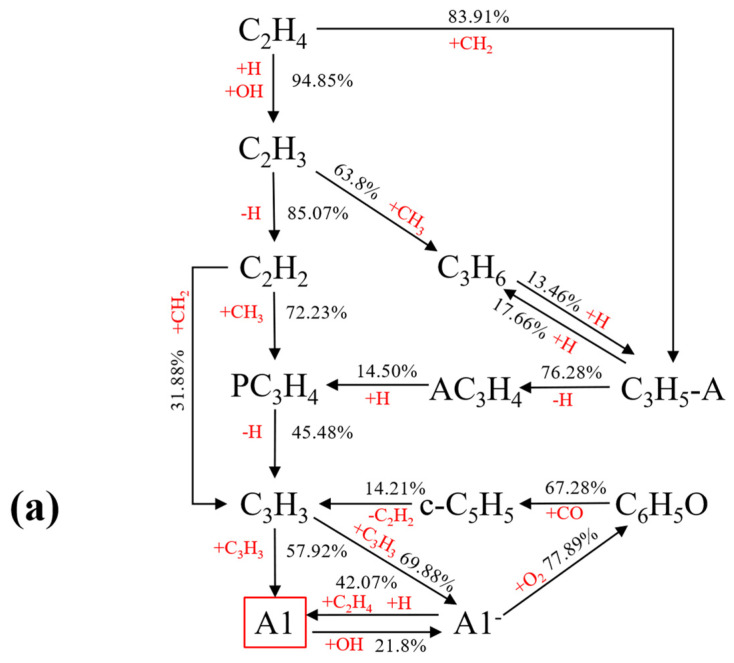

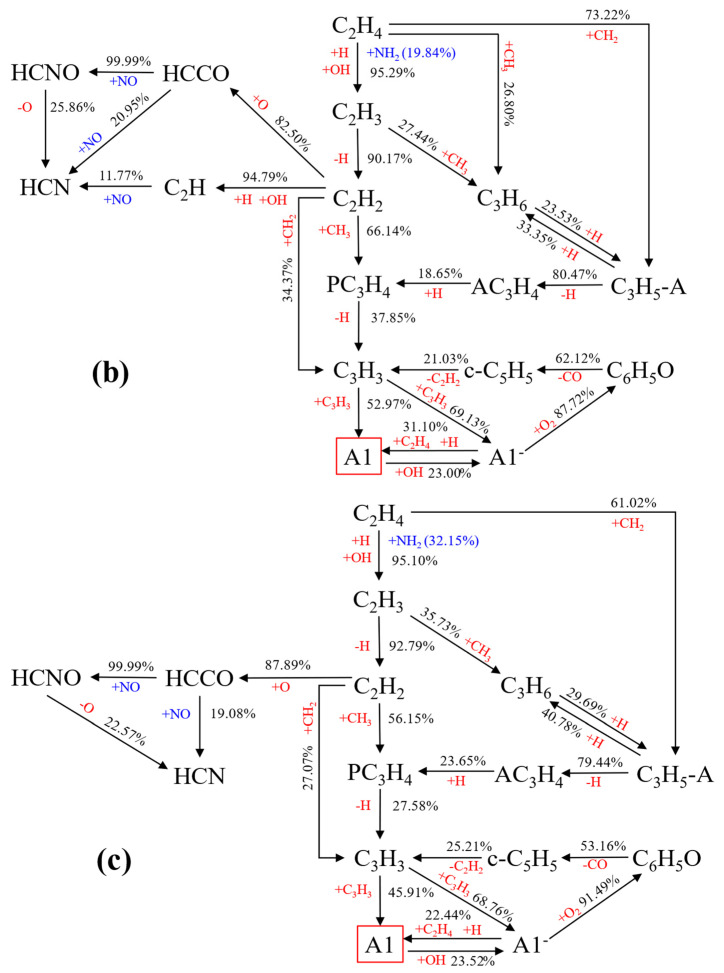
Simplified reaction pathway from C2H4 to A1 with the addition of ammonia: (**a**) 0%; (**b**) 30%; (**c**) 60%.

Figure 8a–c show the simplified reaction pathway from C_2_H_4_ to A1 with 0%, 30%, and 60% ammonia added. The numbers on the pathway indicate the contribution rate of the initial substance to the final product, obtained by integrating the material product rate. During combustion, C_2_H_4_ reacts with H and OH to form C_2_H_3_, which then dehydrogenates to produce C_2_H_2_. This further reacts with CH_3_ to generate propylene diene (PC_3_H_4_). PC_3_H_4_ dehydrogenates to form C_3_H_3_, which then undergoes direct addition to form A1 or A1^−^. A1^−^ is converted to A1 through hydrogenation. Therefore, production of A1 mainly comes from A1^−^ and C_3_H_3_, following this reaction formula:

(3) $$2C_{3}H_{3}=A1$$

(4) $$A1^{-}+H\;\left(+M\right)=A1\;\left(+M\right)$$

(5) $$A1^{-}+C_{2}H_{4}=A1+C_{2}H_{3}$$

In the pathway diagram, the addition of ammonia changes the primary production pathway of A1. Without ammonia, C_2_H_4_ contributes 94.85% to the formation of C_2_H_3_ through its reaction with H and OH. However, with 30% ammonia, NH_2_ reacting with C_2_H_4_ accounts for 19.84% of the total contribution rate, while C_2_H_4_ remains the major contributor at 95.29%. The proportion of NH_2_ capturing the H atom in C_2_H_4_ to generate NH_3_ significantly increases to 32.15% with 60% ammonia. This continuous consumption of H atoms hinders the subsequent formation of A1 and PAH growth, ultimately affecting soot production. The reaction formula is:

(6) $$C_{2}H_{4}+NH_{2}=C_{2}H_{3}+NH_{3}$$

(7) $$NH_{3}+H=NH_{2}+H_{2}$$

With the addition of ammonia, C_2_H_2_ reacts with H, OH, and O to produce HCCO or C_2_H, which subsequently forms HCN in the presence of NO. These reactions not only deplete C_2_H_2_, but also consume carbon atoms, leading to a reduction in the formation of soot.

**Figure 9:**
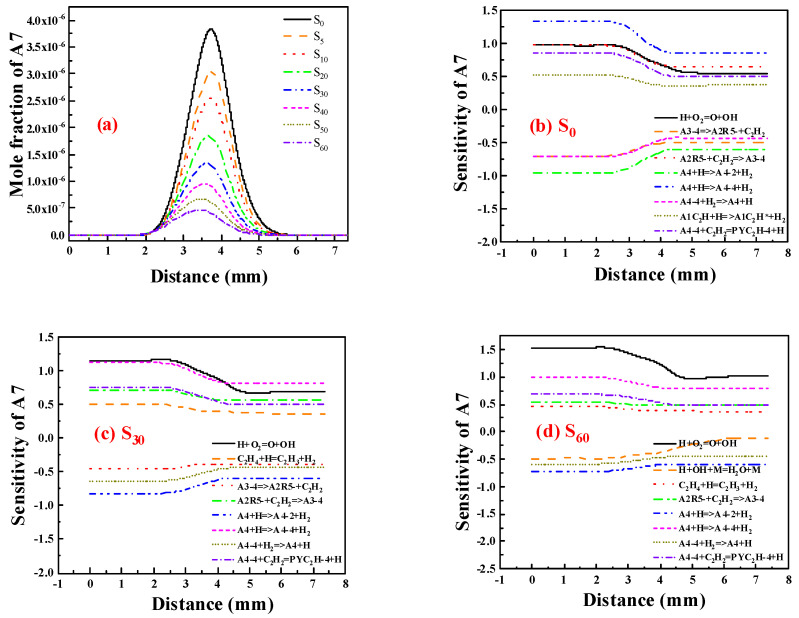
(**a**) Distribution of A7 concentrations after adding different proportions of ammonia; (**b**–**d**) Sensitivity coefficient of A7 with ammonia adding (**b**) 0%; (**c**) 30%; (**d**) 60%.

Figure 9 illustrates the distribution of A7 concentrations and the sensitivity coefficient. The sensitivity coefficient of A7 was changed after adding 0%, 30% and 60% ammonia. It can be seen from the diagram that the largest reaction affecting the formation of A7 without ammonia is the dehydrogenation reaction of A4 to form A4-4, and the largest impact on the consumption of A7 is also the dehydrogenation reaction of A4, but the reaction product is A4-2, in which A4-4 and A4-2 are isomers. This indicates that the role of different isomers in the formation and consumption of A7 is also very different. After adding 30% ammonia, the effect of reaction H + O_2_ = O + OH on the formation of A7 gradually increased, indicating that the addition of ammonia reduced H, which inhibited the formation of PAHs, and the formation of A7 was also inhibited. For 60% ammonia, the effect of the reaction between H and O_2_ on the formation of A7 is further increased, which means that when ammonia is added to the ethylene flame, the greatest impact on PAHs is the change in H concentration. The more ammonia added, the smaller the concentration of H in the flame, and the more the formation of PAHs will be inhibited.

##### Soot Generation Concentration Analysis

**Figure 10:**
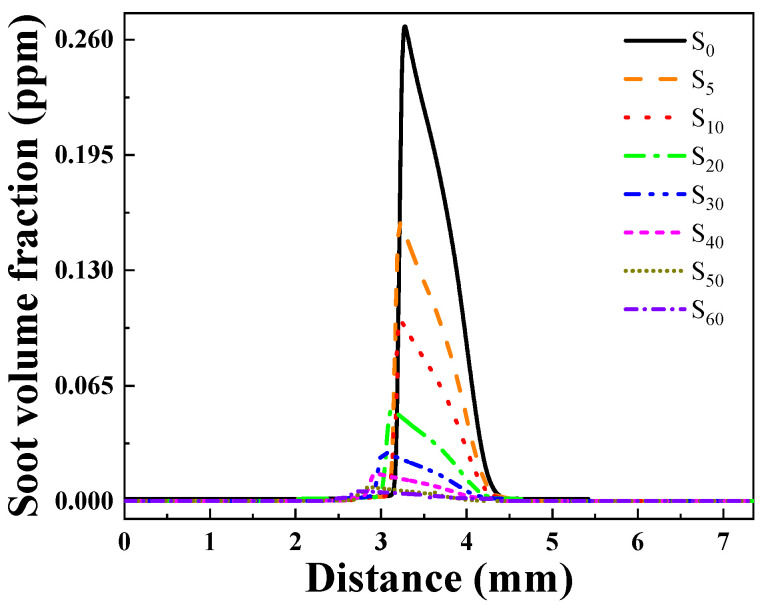
Changes in soot volume fraction after adding different proportions of ammonia.

Figure 10 displays the distribution of soot volume fraction variation with the addition of ammonia. For the case without ammonia, soot particle formation begins at approximately 4.5 mm, and then rapidly decreases towards zero. Following the addition of ammonia, the soot concentration significantly decreases, with the peak values for the S0–S60 flame being 0.275, 0.159, 0.102, 0.0505, 0.0266, 0.0143, 0.00788, and 0.00436 ppm, respectively.

**Figure 11:**
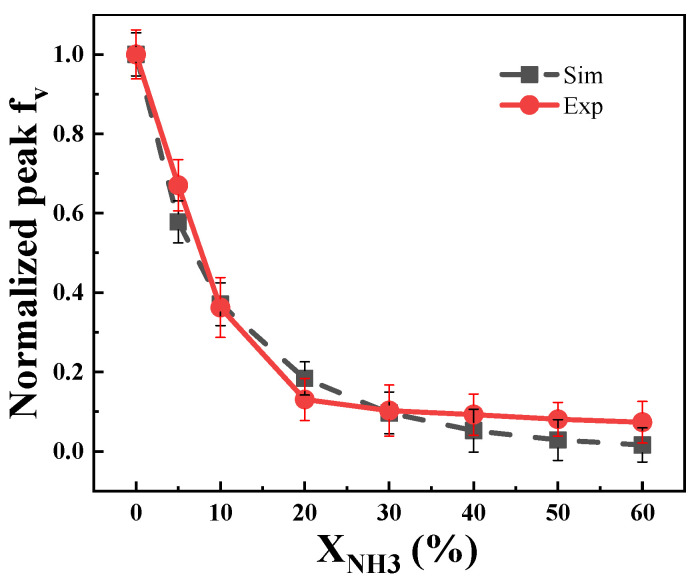
Normalized maximum soot volume fraction by simulation and experiment after ammonia addition.

Figure 11 illustrates the impact of ammonia addition on soot formation in both simulated and experimental flames. The normalized maximum soot volume fraction decreases with increasing ammonia addition, indicating effective inhibition of soot formation. However, the inhibitory effect diminishes when the ammonia addition ratio exceeds 20%. Despite differences, the simulated diffusion flame shows a similar trend to the co-flow diffusion flame under the same ammonia addition conditions. These results confirm that the simulation accurately predicts soot formation after adding ammonia.

#### 2.2.2. The Effect of Ammonia on NOx Formation

##### NOx Generation Concentration Analysis

**Figure 12:**
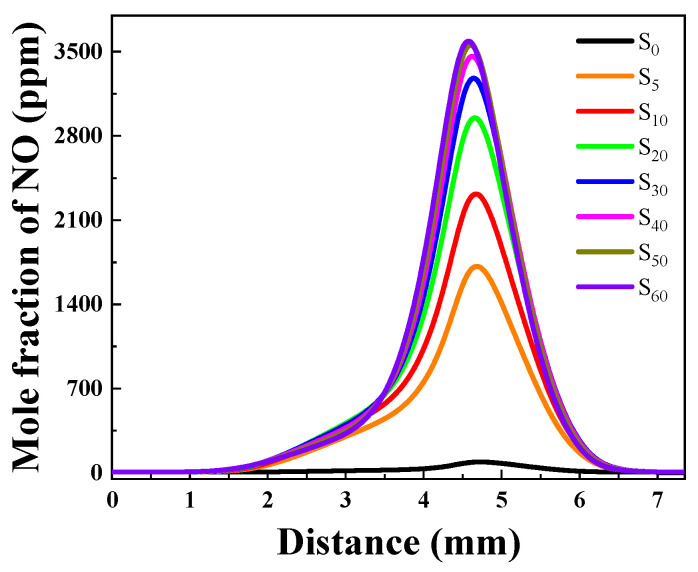
NO concentrations with different proportions of ammonia.

Figure 12 illustrates the variation in NO concentration following the blending of different proportions of ammonia. The maximum value is 1710 ppm when 5% of ammonia is added, significantly higher than the concentration of NO at only 8 ppm without NH_3_. Furthermore, the peak value reaches 3590 ppm for a blend containing 60% ammonia. The trend shows that adding a small amount of ammonia can significantly increase the concentration of NO, with less-noticeable changes in the peak as the ratio of ammonia increases above 30%. This is qualitatively consistent with the previous experimental study of Suresh et al. [40], which reported that the peak NOx value increases by a factor of more than 9 as the NH_3_ content in fuel is increased from 0% to 12.5%. With a further increase in NH_3_ (from 12.5% to 25%), there is still a significant, but relatively more modest, increase in NOx.

##### ROP Analysis of NO

**Figure 13:**
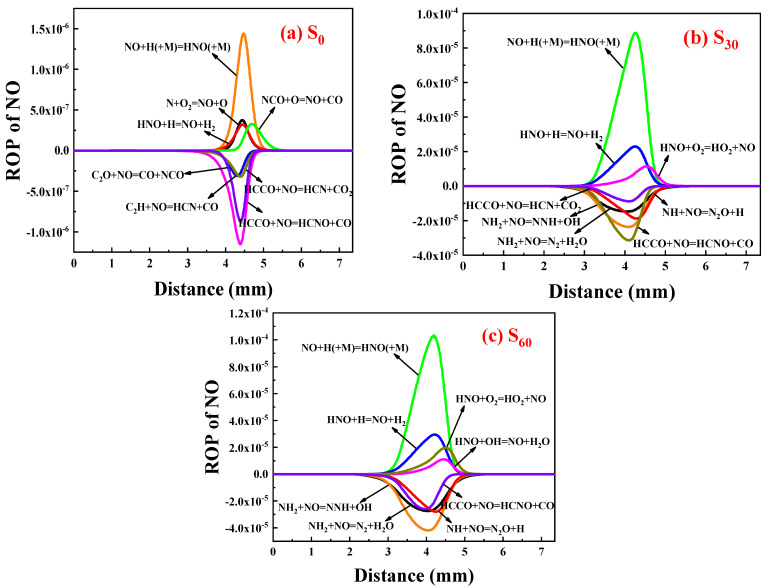
ROP of NO reaction after adding ammonia: (**a**) 0%; (**b**) 30%; (**c**) 60%.

Figure 13 displays the ROP distribution of each NO reaction after doping with 0%, 30%, and 60% ammonia. It shows the top eight absolute values of the reaction rate magnitude, where positive values indicate NO generation and negative values indicate NO consumption.

It can be seen that doping with ammonia increases the NO generation reaction rate. NO formation is due to the reaction via HNO as well as NH_3_ combustion in NH_3_/air. In the C_2_H_4_ flame, the most important NO formation channel was HNO (+M) = NO + H (+M) and the second was N + O_2_ = NO + O. In the C_2_H_4_-NH_3_ flame, the reaction rate of HNO (+M) = NO + H (+M) increases by two orders of magnitude, which was mainly responsible for the increase in NO formation. In addition, HNO + H = NO + H_2_, HNO + O_2_ = HO_2_ + NO and HNO + OH = NO + H_2_O also played important roles in the increase in NO formation. The most important precursor participating in NO formation was N radicals and HNO in C_2_H_4_ flame, while it was HNO in C_2_H_4_-NH_3_ flame. Compared to C_2_H_4_/air combustion, the rate of NO consumption increases by a factor of 30 and 40 for 30% and 60% ammonia doping, respectively. The reaction with the fastest rate of NO consumption without ammonia is HCCO + NO = HCNO + CO, and the reaction with the fastest rate of NO consumption with 60% ammonia is NH_2_ + NO = NNH + OH. This is due to the fact that as the amount of ammonia doped continues to increase, the ammonia that is not oxidized in time in the flame will begin to reduce some of the NO produced.

##### Reaction Path Analysis of NO

**Figure 14:**
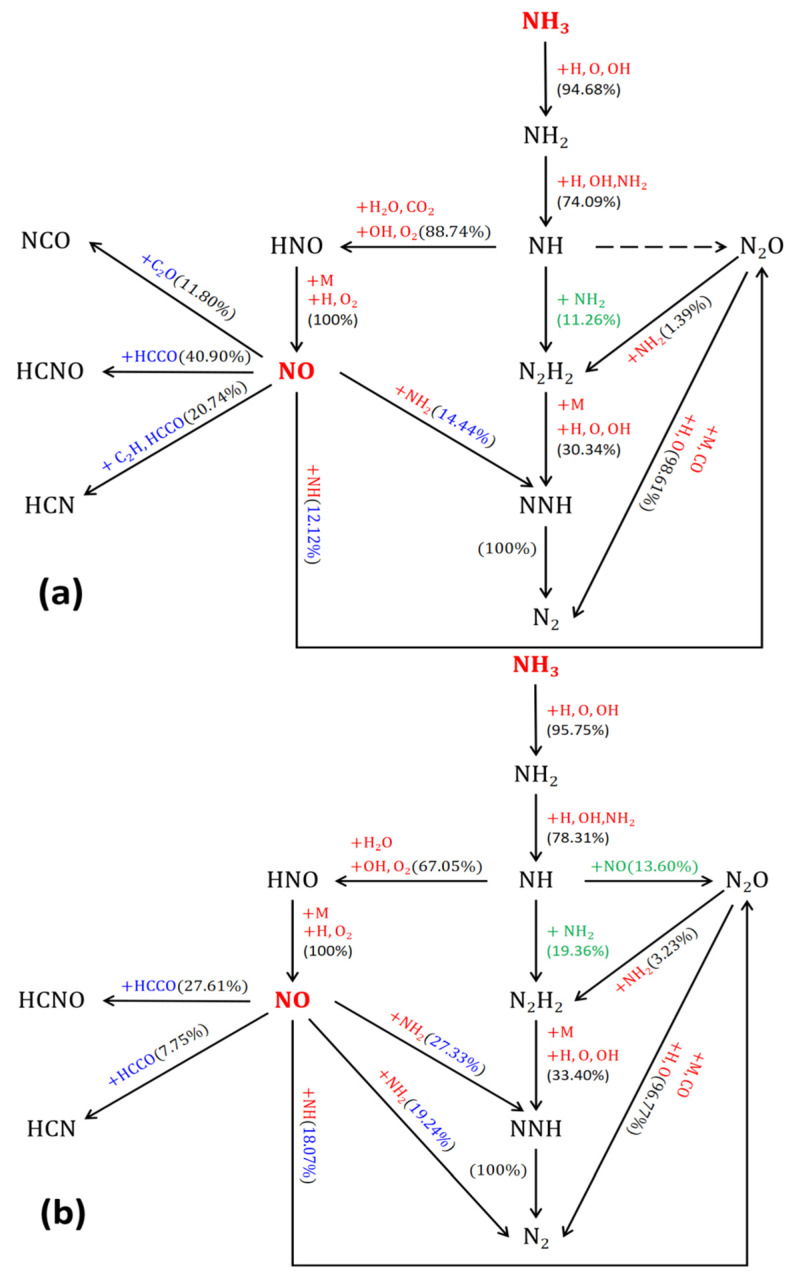

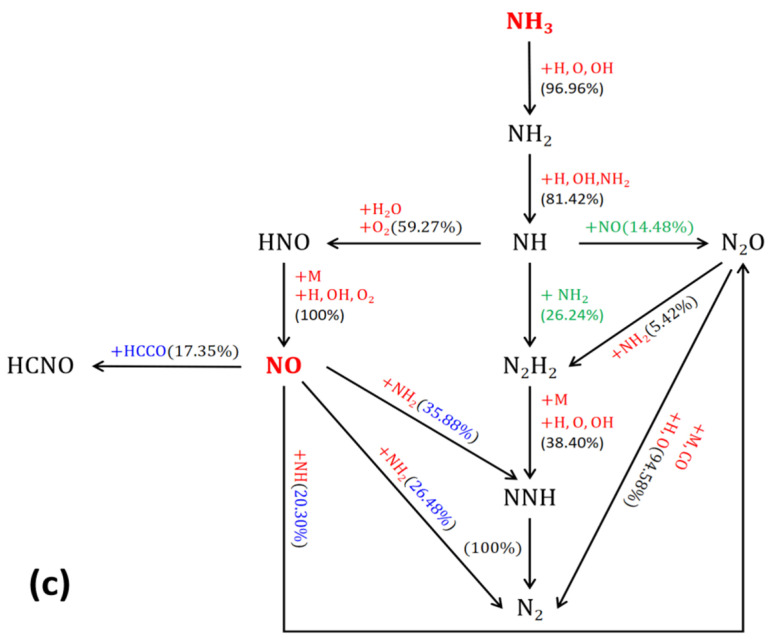
Simplified reaction pathway from NH_3_ to NO with the addition of ammonia: (**a**) 10%; (**b**) 30%; (**c**) 60%.

In hydrocarbon flames, the addition of NH_3_ in the fuel stream led to a significant increase in NO levels in C_2_H_4_-NH_3_ flame [41]. Figure 14a–c illustrate simplified reaction paths for NO generation with 10%, 30%, and 60% ammonia blending, respectively. The numbers on the paths indicate the contribution of each substance to the generation of NO, obtained by integrating substance product rates and comparing them.

From the path diagram, it is clear that the ammonia blending rate has less effect on the main path of NO generation. NH_3_ generates the free radicals NH_2_ and NH under the impact of H radicals, oxygen radicals, and hydroxyl OH. The free radical NH forms the intermediate product HNO under the action of hydroxyl OH and oxygen, which is then oxidized to the product NO. It is worth noting that the free radical NH can also form N_2_H_2_ and N_2_O under the action of the free radicals NH_2_ and NO, respectively, finally forming N_2_. When the ammonia doping rate is higher, there is a higher chance of NH_3_ being oxidized to N_2_. Additionally, when the ammonia mixing rate is higher, generated NO is more likely to be reduced to N_2_ again under the action of the free radicals NH_2_ and NH. This leads to a significant increase in NO concentration when a small amount of ammonia is mixed; however, this trend becomes less obvious as the mixing ratio increases.

## 3. Materials and Methods

### 3.1. Experimental Setting

#### 3.1.1. Experimental System and Setting

**Figure 15:**
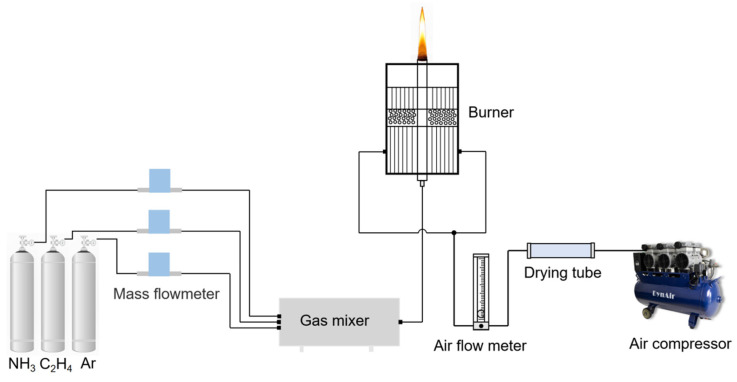
Experimental combustion system.

Figure 15 shows the combustion system structure used. The burner is based on the Gülder [42] burner commonly used in diffusion flame research, with an inner diameter of 10 mm at the outlet of the fuel pipe and an outer diameter of 14 mm, and an annular oxidizer sleeve consisting of porous metal foam outside the fuel pipe to provide a smooth and uniform flow of air; the sleeve has an inner diameter of 88 mm and a wall thickness of 8 mm; more detailed information has been described in Refs. [43,44,45]. Ethylene and ammonia (purity > 99.99%) were used as fuel, and argon (purity > 99.99%) was used as diluent. The corresponding mass flowmeters were used to control the fuel flow.

Table 1 shows the experimental parameters in this study. C_2_H_4_, NH_3_, and Ar (purity > 99.99%) were used as fuel and dilution gas. During the experiment, the C_2_H_4_ flow rate was fixed at 180 mL/min, and different proportions of NH_3_ were used instead of Ar to eliminate the effect of dilution. The experimental NH_3_ mixing ratios were set at 0%, 5%, 10%, 20%, 30%, 40%, 50%, and 60%, which were marked as X_0_, X_5_, X_10_, X_20_, X_30_, X_40_, X_50,_ and X_60,_ respectively. The compressor supplies the oxidizer for combustion at a fixed flow rate of 100 L/min.

#### 3.1.2. Measurement of Soot Volume Fraction

The soot volume fraction was measured using light extinction (LE) technology, and the soot volume fraction (SVF, *f_V_*) can be calculated using the following formula:

(8) $$f_{V}=\frac{K_{ext}\lambda }{6\pi E\left(m\right)}$$

where the *f_V_* is the soot volume fraction; λ is the wavelength of light; *E*(*m*) is a function of the complex refractive index of soot particles, which is only related to the physical characteristics of the soot particles (in this work *E*(*m*) = 0.26 [46]); and K*_ext_* is the extinction coefficient of the particles, which can be calculated using the Abel inversion algorithm [47]. The uncertainty of *E*(*m*) does not exceed 10%, and the overall uncertainty of the soot volume fraction measurement is <30% [48].

**Figure 16:**
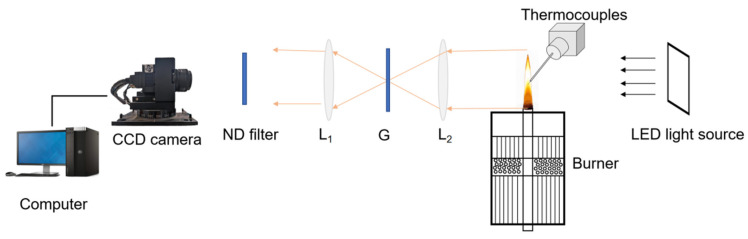
The LE system setup. L_1_ and L_2_: plano-convex lenses 1 and 2; G: grating with a pinhole.

The soot concentration was deduced by analyzing and processing the light intensity. A light source was used to emit light of a specific wavelength to the surface of the measured object. As shown in Figure 16, the light extinction measurement system consists of an LED light source that emits collimated light, two plano-convex lenses (L_1_ and L_2_), a pinhole grating (G), a neutral density (ND) filter (15% transmission), a CCD camera, and a computer. The CCD camera array size is 964 × 92 pixels with a pixel size of 200 μ, and a set speed of 30 frames per second and an exposure time of 5000 μs were used in the experiment. The light source is a flat LED light-emitting panel that produces a stable parallel light and can completely cover the entire flame height to ensure accuracy of the measurement results. L_1_ and L_2_ are a pair of identical plano-convex lenses whose surfaces are opposite each other. The light passing through the flame is focused by lens L_2_ at the small hole grating (G). As light passes through the L_1_ and ND filters, LED light and flame light are captured by the CCD camera. More information has been mentioned in previous studies [36,38]. In order to ensure the accuracy of the experiment, all experiments were repeated at least three times.

#### 3.1.3. Measurement of NOx

**Figure 17:**
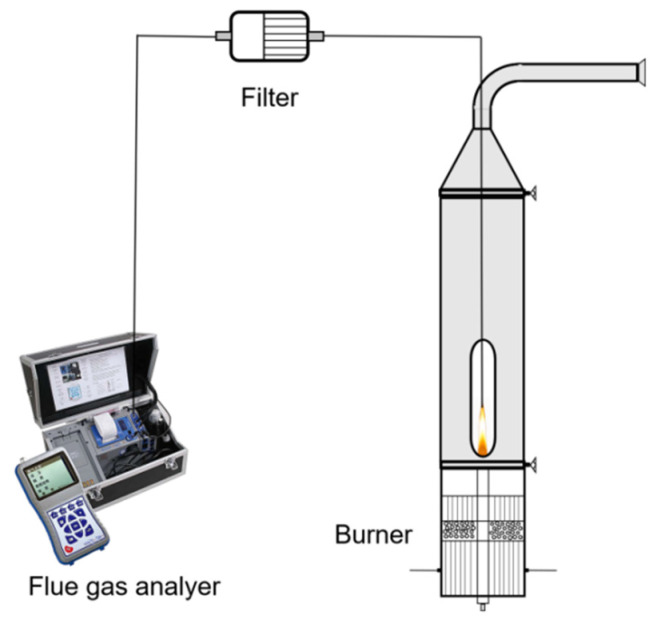
The schematic diagram of the NOx measurement system.

The flue gas sampling system is shown in Figure 17. The sampling tube is made of stainless steel with a diameter of 2 mm, and the flue gas analysis is carried out using an ECOM-J2KN flue gas analyer made in Iserlohn, Germany, capable of measuring NO, O_2_, CO, and other gases. The O_2_ measurement concentration range is 0–21% vol, with an accuracy of 0.2 vol and a resolution of 0.1%. The NO concentration measurement range is 0–2000 ppm, with an upper limit of 5000 ppm, a measurement error of 5 ppm, and a resolution of 1 ppm. The CO measurement range is 0–4000 ppm, with an upper limit of 10,000 ppm, an accuracy of 5 ppm, and a resolution of 1 ppm. Therefore, the flue gas analyzer meets the requirements of this experiment.

### 3.2. Simulation Methods

The opposed flow diffusion flame model (OPPDIF) in the CHEMKIN software [49] was used to simulate the soot formation characteristics in this work [26,50,51]. The inlet temperature produced by the fuel and oxidizer is the same as the inlet temperature produced by the co-flow diffusion flame studied in the experiment. The inlet gas velocity of both nozzles was set to 15 cm/s (298 K) and the pressure was set to 1 atm. The distance between the two nozzles is 7.35 mm. The KM2 mechanism [52] was chosen as the main mechanism for elemental carbon reactions in the combustion simulation, but the reaction rate of H was found to be too rapid after ammonia doping in the calculation, so the reaction rate of some H reactions was modified in this paper according to the results of the sensitivity analysis. The Glarborg mechanism [53] was chosen for the nitrogen reaction, and it was found that the Glarborg–GRI 3.0 mechanism could be closer to the experimental results by adding some reaction steps of the GRI 3.0 mechanism [54]. The soot model used was the soot surface reaction model developed by Wang et al. [55], which has 36 species, considers eight PAH molecules, and has a more complete description of soot nucleation. The soot surface growth reaction is based on the modified HACA mechanism, and CH_3_, C_3_H_3_, and C_2_H were also included in the hydrogen extraction reaction in addition to H.

**Figure 18:**
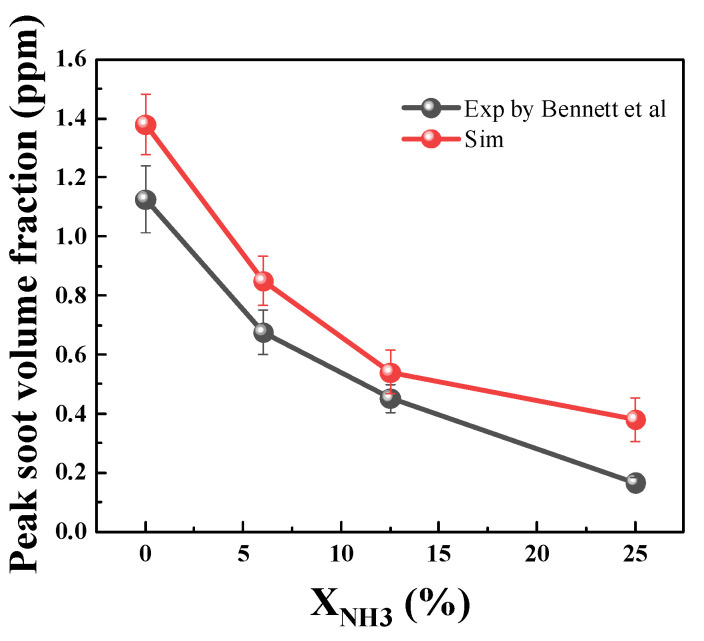
Comparison of simulation results and experimental results [22].

The accuracy of the mechanism needs verification upon completion of its integration. Figure 18 shows the comparison of the simulation results and the experimental results of Bennett et al. [22] using the same ammonia doping ratios. In the figure, a large difference between the simulation and experimental results is observed for the undoped ammonia condition, which gradually decreases after ammonia doping. The upper limit of error between the simulated and experimental results for unadulterated ammonia is 13.9%, but there is also a larger experimental error range at this time. The difference between the simulation with 12.5% ammonia and the experimental results is smaller, at 9.2%, indicating better simulation results at this point. At 25% ammonia doping, there is a larger difference between the experimental and simulated results. However, considering that soot concentration is very low at this time, the absolute change in soot concentration is even lower compared to the lower-ammonia-percentage doping conditions. In conclusion, it can be stated that both the reaction mechanism and soot growth model effectively reflect actual experiment trends.

## 4. Conclusions

Soot and NOx are two primary pollutants formed during the combustion of hydrocarbon fuels. Understanding the mechanisms of soot and NOx formation is of great significance in guiding practical fuel combustion due to the need to control pollutant formation. This study investigates the effect of ammonia addition on the mechanisms of soot and NOx formation of ammonia/ethylene flames. The spatial soot volume fraction distribution, the variation of NOx concentrations, and the chemical impact of ammonia on the formation of soot and NOx are analyzed in detail. The main conclusions obtained are as follows:

1. Ammonia doping reduces soot formation in ethylene flames. When the doping ratio of ammonia is below 20%, there is a strong inhibitory effect on the soot concentration in the flame, and the inhibitory effect on soot changes less for cases with more than 30% added ammonia.
2. A higher concentration of NOx is generated in the initial part of the flame, and the lower the ammonia ratio, the higher the concentration of NOx. As the combustion process progresses, the concentration of NOx initially decreases and then subsequently increases rapidly. With higher ammonia ratios, there is a higher concentrations of NOx emissions.
3. Ammonia doping reduces the mole fractions of C_2_H_2_, C_3_H_3_, PC_3_H_4_, and C_4_H_4_ responsible for the first aromatic ring formation and larger PAH growth, thus leading to the reduction of A1–A4 formation. In addition, sensitivity and reaction pathway analysis of A1 formation indicate that NH_3_ addition contributes to the nitrogen–hydrocarbon interaction, which hinders the HACA process and inhibits the generation of soot.
4. The ammonia blending rate has little effect on the main pathway of NO generation, but the higher the amount of ammonia, the higher the chance of NH_3_ being oxidized to N_2_, and the more likely it is that the generated NO will be reduced to N_2_ again by the action of the free radicals NH_2_ and NH.

## Figures and Tables

**Table 1:** Operating conditions.

Case	NH_3_ Ratio	NH_3_ Flow Rate (mL/min)	C_2_H_4_ Flow Rate (mL/min)	Ar Flow Rate (mL/min)	Air Flow Ate (L/min)
X_0_	0%	0	180	270	100
X_5_	5%	22.5	180	247.5	100
X_10_	10%	45	180	225	100
X_20_	20%	90	180	180	100
X_30_	30%	135	180	135	100
X_40_	40%	180	180	90	100
X_50_	50%	225	180	50	100
X_60_	60%	270	180	0	100

## Data Availability

The original contributions presented in the study are included in the article, further inquiries can be directed to the corresponding author.

## References

[1] Outlook. https://www.irena.org/Energy-Transition/Outlook.

[2] Dimitriou P., Javaid R. (2020). A review of ammonia as a compression ignition engine fuel. Int. J. Hydrogen Energy.

[3] Elbaz A.M., Wang S., Guiberti T.F., Roberts W.L. (2022). Review on the recent advances on ammonia combustion from the fundamentals to the applications. Fuel Commun..

[4] (2021). Ammonia Technology Roadmap.

[5] Valera-Medina A., Amer-Hatem F., Azad A.K., Dedoussi I.C., De Joannon M., Fernandes R.X., Glarborg P., Hashemi H., He X., Mashruk S. (2021). Review on ammonia as a potential fuel: From synthesis to economics. Energy Fuels.

[6] Chai W.S., Bao Y., Jin P., Tang G., Zhou L. (2021). A review on ammonia, ammonia-hydrogen and ammonia-methane fuels. Renew. Sustain. Energy Rev..

[7] Valera-Medina A., Xiao H., Owen-Jones M., David W.I.F., Bowen P.J. (2018). Ammonia for power. Prog. Energy Combust. Sci..

[8] Zamfirescu C., Dincer I. (2008). Using ammonia as a sustainable fuel. J. Power Sources.

[9] Kobayashi H., Hayakawa A., Somarathne K.D.K.A., Okafor E.C. (2019). Science and technology of ammonia combustion. Proc. Combust. Inst..

[10] Zhang R., Chen L., Wei H. (2022). Understanding the difference in combustion and flame propagation characteristics between ammonia and methane using an optical SI engine. Fuel.

[11] Nozari H., Karabeyoğlu A. (2015). Numerical study of combustion characteristics of ammonia as a renewable fuel and establishment of reduced reaction mechanisms. Fuel.

[12] Ariemma G.B., Sorrentino G., Ragucci R., de Joannon M., Sabia P. (2022). Ammonia/Methane combustion: Stability and NOx emissions. Combust. Flame.

[13] Ku J.W., Choi S., Kim H.K., Lee S., Kwon O.C. (2018). Extinction limits and structure of counterflow nonpremixed methane-ammonia/air flames. Energy.

[14] Hashimoto G., Hadi K., Xia Y., Hamid A., Hashimoto N., Hayakawa A., Kobayashi H., Fujita O. (2021). Turbulent flame propagation limits of ammonia/methane/air premixed mixture in a constant volume vessel. Proc. Combust. Inst..

[15] Tang R., Xu Q., Pan J. (2022). An experimental and modeling study of ammonia oxidation in a jet stirred reactor. Combust. Flame.

[16] Awad O., Zhou B., Harrath K., Kadirgama K. (2022). Characteristics of NH_3_/H_2_ blend as carbon-free fuels: A review. Int. J. Hydrogen Energy.

[17] Deng Q., Ying Y., Liu D. (2022). Detailed chemical effects of ammonia as fuel additive in ethylene counterflow diffusion flames. Int. J. Hydrogen Energy.

[18] Li Y., Zhang Y., Yang G., Fuentes A., Han D., Huang Z., Lin H. (2022). A comparative study on PAH characteristics of ethanol and ammonia as fuel additives in a premixed flame. J. Energy Inst..

[19] Yan Z., Yang Y., Li Q., Yan Y., Tian Z., Song C., Huang Z. (2023). Study on effects of NH_3_ and/or H_2_ addition on the characteristics of soot formation and gas emissions in a laminar ethylene diffusion flame. Fuel Process. Technol..

[20] He J., Ying Y., Chen M., Liu D. (2022). Soot formation characteristics in hybrid pyrolysis of zero-carbon fuel ammonia and ethylene mixtures. Front. Energy Res..

[21] Chen Z., Huang X., Huang C., Yang Y., Yang H., Zhang J., Huang T. (2022). High atmospheric wet nitrogen deposition and major sources in two cities of Yangtze River Delta: Combustion-related NH_3_ and non-fossil fuel NOx. Sci. Total Environ..

[22] Bennett A.M., Liu P., Li Z., Kharbatia N.M., Boyette W., Masri A.R., Roberts W.L. (2020). Soot formation in laminar flames of ethylene/ammonia. Combust. Flame.

[23] Zhou M., Yan F., Ma L., Jiang P., Wang Y., Ho Chung S. (2022). Chemical speciation and soot measurements in laminar counterflow diffusion flames of ethylene and ammonia mixtures. Fuel.

[24] Steinmetz S.A., Ahmed H.A., Boyette W.R., Dunn M.J., Roberts W.L., Masri A.R. (2022). Effects of ammonia and hydrogen on the sooting characteristics of laminar coflow flames of ethylene and methane. Fuel.

[25] Liu Y., Cheng X., Li Y., Qiu L., Wang X., Xu Y. (2021). Effects of ammonia addition on soot formation in ethylene laminar diffusion flames. Fuel.

[26] Ren F., Cheng X., Gao Z., Huang Z., Zhu L. (2022). Effects of NH_3_ addition on polycyclic aromatic hydrocarbon and soot formation in C_2_H_4_ co-flow diffusion flames. Combust. Flame.

[27] Boyette W.R., Macfarlane A.R.W., Steinmetz S.A., Dunn M.J., Roberts W.L., Masri A.R. (2023). On the combined effects of compositional inhomogeneity and ammonia addition to turbulent flames of ethylene. Proc. Combust. Inst..

[28] Zhang K., Xu Y., Liu Y., Wang H., Liu Y., Cheng X. (2022). Effects of ammonia addition on soot formation in ethylene laminar diffusion flames. Part 2. Further insights into soot inception, growth and oxidation. Fuel.

[29] Da Rocha R.C., Costa M., Bai X.-S. (2019). Chemical kinetic modelling of ammonia/hydrogen/air ignition, premixed flame propagation and NO emission. Fuel.

[30] Xiao H., Lai S., Valera-Medina A., Li J., Liu J., Fu H. (2020). Study on counterflow premixed flames using high concentration ammonia mixed with methane. Fuel.

[31] Abián M., Peribáñez E., Millera Á., Bilbao R., Alzueta M.U. (2014). Impact of nitrogen oxides (NO, NO_2_, N_2_O) on the formation of soot. Combust. Flame.

[32] Zhao Z., Zhang Z., Zha X., Gao G., Mao W., Wu F., Li X. (2023). Fuel-NO formation mechanism in MILD-oxy combustion of CH_4_/NH_3_ fuel blend. Fuel.

[33] Shi H., Liu S., Zou C., Dai L., Li J., Xia W., Yang J., Luo J., Li W. (2022). Experimental study and mechanism analysis of the NOx emissions in the NH3 MILD combustion by a novel burner. Fuel.

[34] Montgomery M.J., Kwon H., Dreyer J.A.H., Xuan Y., McEnally C.S., Pfefferle L.D. (2021). Effect of ammonia addition on suppressing soot formation in methane co-flow diffusion flames. Proc. Combust. Inst..

[35] Guo H., Smallwood G.J. (2007). The interaction between soot and NO formation in a laminar axisymmetric coflow ethylene/air diffusion flame. Combust. Flame.

[36] Wang Q., Legros G., Bonnety J., Morin C. (2017). Experimental characterization of the different nitrogen dilution effects on soot formation in ethylene diffusion flames. Proc. Combust. Inst..

[37] Gu M., Chu H., Liu F. (2016). Effects of simultaneous hydrogen enrichment and carbon dioxide dilution of fuel on soot formation in an axisymmetric coflow laminar ethylene/air diffusion flame. Combust. Flame.

[38] Gu M., Liu F., Consalvi J.L., Gülder Ö.L. (2021). Effects of pressure on soot formation in laminar coflow methane/air diffusion flames doped with n-heptane and toluene between 2 and 8 atm. Proc. Combust. Inst..

[39] Daca A.E., Gülder Ö.L. (2017). Soot formation characteristics of diffusion flames of methane doped with toluene and n-heptane at elevated pressures. Proc. Combust. Inst..

[40] Suresh R., Kalvakala K.C., Aggarwal S.K. (2024). A numerical study of NOx and soot emissions in ethylene-ammonia diffusion flames with oxygen enrichment. Fuel.

[41] Yang Y., Zheng S., Sui R., Lu Q. (2023). Impact of ammonia addition on soot and NO/N_2_O formation in methane/air co-flow diffusion flames. Combust. Flame.

[42] Gülder Ö.L., Snelling D.R., Sawchuk R.A. (1996). Influence of hydrogen addition to fuel on temperature field and soot formation in diffusion flames. Symp. Combust..

[43] Zhang F., Li S., Liu Q., Sun J., Wei X., Gu M., Wang Y., Huang X. (2023). Effect of ammonia on the soot surface characteristics in ammonia/ethylene co-flow diffusion flames. Fuel.

[44] Lin Y., Zhu B., Chen J., Wu J., Lu K., Gu M., Chu H. (2020). Study of soot functional groups and morphological characteristics in laminar coflow methane and ethylene diffusion flames with hydrogen addition. Fuel.

[45] Zhu Y., Wu J., Zhu B., Wang Y., Gu M. (2021). Experimental study on the effect of hydrogen addition on methane/ethylene diffusion flame soot formation based on light extinction measurement. Energy Rep..

[46] Smyth K.C., Shaddix C.R. (1996). The elusive history of m = 1.57 − 0.56i for the refractive index of soot. Combust. Flame.

[47] Snelling D.R., Thomson K.A., Smallwood G.J., Gülder Ö.L. (1999). Two-dimensional imaging of soot volume fraction in laminar diffusion flames. Appl. Opt..

[48] Wang Y., Gu M., Gao Y., Liu X., Lin Y. (2020). An experimental and numerical study of soot formation of laminar coflow H_2_/C_2_H_4_ diffusion flames in O_2_-CO_2_ atmosphere. Combust. Flame.

[49] Ansys Ansys Chemkin-Pro|Chemical Kinetics Simulation Software. https://www.ansys.com/products/fluids/ansys-chemkin-pro.

[50] Gao Z., Zhu L., Zou X., Liu C., Tian B., Huang Z. (2019). Soot reduction effects of dibutyl ether (DBE) addition to a biodiesel surrogate in laminar coflow diffusion flames. Proc. Combust. Inst..

[51] Zhang T., Zhao L., Thomson M.J. (2017). Effects of n-propylbenzene addition to n-dodecane on soot formation and aggregate structure in a laminar coflow diffusion flame. Proc. Combust. Inst..

[52] Wang Y., Raj A., Chung S.H. (2013). A PAH growth mechanism and synergistic effect on PAH formation in counterflow diffusion flames. Combust. Flame.

[53] Glarborg P., Miller J.A., Ruscic B., Klippenstein S.J. (2018). Modeling nitrogen chemistry in combustion. Prog. Energy Combust. Sci..

[54] University of California-Berkeley GRI-Mech 3.0. http://combustion.berkeley.edu/gri-mech/version30/text30.html.

[55] Wang Y., Raj A., Chung S.H. (2015). Soot modeling of counterflow diffusion flames of ethylene-based binary mixture fuels. Combust. Flame.

